# Targeting Oncoimmune Drivers of Cancer Metastasis

**DOI:** 10.3390/cancers13030554

**Published:** 2021-02-01

**Authors:** Chie Kudo-Saito, Yukinori Ozaki, Hiroshi Imazeki, Hideyuki Hayashi, Jun Masuda, Hiroki Ozawa, Yamato Ogiwara

**Affiliations:** Department of Immune Medicine, National Cancer Center Research Institute, Tokyo 104-0045, Japan; yukozaki@ncc.go.jp (Y.O.); himazeki@ncc.go.jp (H.I.); hidhayas@ncc.go.jp (H.H.); jmasuda@ncc.go.jp (J.M.); hiozawa@ncc.go.jp (H.O.); yaogiwar@ncc.go.jp (Y.O.)

**Keywords:** metastasis, EMT, cancer stem, polyploidization, immunity, immunotherapy, immune checkpoint, immunosuppression, immune exhaustion, immune dysfunction

## Abstract

**Simple Summary:**

Despite great advances in the detailed profiling of tumor cells and the development of therapeutic agents, cancer metastasis is still a big hurdle in the treatment of cancer patients. This is possibly because tumor cells plastically evolve through interplay with the host environment, including stromal cells, vascular cells, and immune cells. The reciprocal evolution among the numerous components further increases the heterogeneity and complexity in both tumor cells and the host, leading to refractory cancer. It is important to better understand the entire metastatic cascade and the practical implementations targeting the oncoimmune drivers in the mechanisms. This review aims to boost the idea to break down the vicious spiral of the tumor–immunity aggravation more efficiently by combining some different agents in clinical settings.

**Abstract:**

Residual metastasis is a major cause of cancer-associated death. Recent advances in understanding the molecular basis of the epithelial–mesenchymal transition (EMT) and the related cancer stem cells (CSCs) have revealed the landscapes of cancer metastasis and are promising contributions to clinical treatments. However, this rarely leads to practical advances in the management of cancer in clinical settings, and thus cancer metastasis is still a threat to patients. The reason for this may be the heterogeneity and complexity caused by the evolutional transformation of tumor cells through interactions with the host environment, which is composed of numerous components, including stromal cells, vascular cells, and immune cells. The reciprocal evolution further raises the possibility of successful tumor escape, resulting in a fatal prognosis for patients. To disrupt the vicious spiral of tumor–immunity aggravation, it is important to understand the entire metastatic process and the practical implementations. Here, we provide an overview of the molecular and cellular links between tumors’ biological properties and host immunity, mainly focusing on EMT and CSCs, and we also highlight therapeutic agents targeting the oncoimmune determinants driving cancer metastasis toward better practical use in the treatment of cancer patients.

## 1. Introduction

Residual metastasis is a major cause of cancer-associated death. The epithelial–mesenchymal transition (EMT) is a pivotal biological program in cancer metastasis and confers high mobility, invasive, and metastatic properties, and anti-apoptotic dormancy on tumor cells [1,2], although several studies have reported the dispensability of EMT [3,4]. Great progress in the molecular profiling of cancer EMT and the related cancer-initiated cells or cancer stem cells (CSCs) has defined the detailed characteristics, and the advances in understanding the molecular basis of EMT have revealed the landscapes of cancer metastasis, leading to the development of numerous treatments targeting the phenotypes and signaling pathways [5].

To date, however, cancer metastasis is still a threat to cancer patients due to the high frequency and the high mortality in clinical settings. An important point is that the EMT is only a part of the metastatic cascade. The disseminated dormant tumor cells must reawaken to grow in the metastatic secondary site, possibly through the reverse process of EMT, the so-called mesenchymal-to-epithelial transition (MET), and/or polyploidy that giant cells rapidly generate progeny cells with genomic instability in response to treatment stress [6,7,8].

On the other hand, accumulating evidence suggests that epigenetic modification by the host environment, which is composed of numerous components, including stromal cells, vascular cells, and immune cells, impacts on every step of the metastatic cascade: tumor development, dissemination, invasion, intravasation, extravasation, colonization, and survival [9,10]. The reciprocal evolution among these factors could massively increase the oncoimmunological heterogeneity and complexity in the host, and raises the possibility of cancer metastasis. Advanced technologies have revealed the landscape of tumor-supportive immunity, and a variety of immunotherapeutics have been developed in clinical settings [11].

The most outstanding effort is targeting immune inhibitory checkpoint pathways, which are innate brakes on anti-tumor immune responses. Immune checkpoint inhibitors (ICIs) have been considered to be a promising immunotherapeutic to knockout the immune underpinnings and have shown great therapeutic efficacies, particularly long-lasting durable responses, even in patients with advanced and metastatic cancer [12]. However, the response rate remains relatively low in many cases since most patients eventually show acquired resistance to the treatment even if they respond in the beginning [12]. Adverse events, including autoimmunity [13] and hyperprogression [14,15], are frequently seen in the treated patients. Hyperprogression is a rapid deterioration of the tumor growth and metastasis in patients shortly after treatment with ICIs, although the incidence varies between 4% and 29% of all responses using various defining criteria [14,15]. Thus, more effective but less toxic treatments are still needed in clinical settings.

To disrupt the vicious spiral of the tumor–immunity aggravation leading to cancer metastasis, it is important to better understand both the entire metastatic cascade and the clinical implementation. In this review, we highlight the molecular and cellular links between the tumor biology and host immunity on the basis of cancer metastasis and provide an overview of the therapeutic agents targeting the oncoimmune determinants driving cancer metastasis toward better practical use in the treatment of cancer patients.

## 2. Initiation of Tumor Escape through EMT

**Figure 1:**
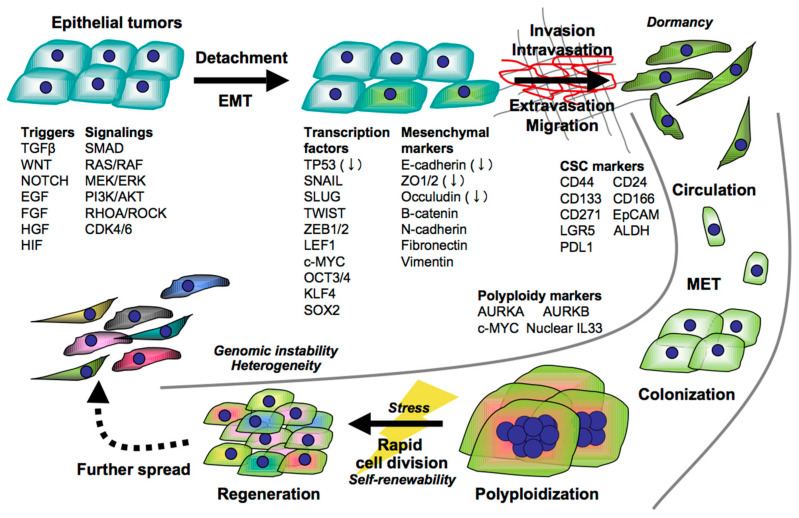
Tumor evolutional transformation through epithelial–mesenchymal transition (EMT), the reverse process of EMT (mesenchymal-to-epithelial transition, MET), and polyploidization toward successful metastasis. The molecules designated are representative molecules that are induced and enhanced in the tumor cells, except decreased molecules with a down arrow.

Epithelial tumor cells adhere to each other via tight junctions, but sometimes transform into mesenchymal types with low adhesive but high invasive properties in response to extrinsic stimuli, such as hypoxia and inflammation mediated by numerous factors, including WNT, NOTCH, transforming growth factor beta (TGFβ), epidermal growth factor (EGF), fibroblast growth factor (FGF), hepatocyte growth factor (HGF), and hypoxia inducible factor (HIF) within the tumor microenvironment [1,2]. This biological program is widely known as EMT (Figure 1).

TGFβ stands out as a master regulator of cancer EMT, and the canonical TGFβ-SMAD pathway plays a key role in the EMT program in cooperation with other signaling pathways, such as PI3K/AKT, ERK/MAPK, PAR6, ras homolog family member A (RHOA), and Rho-associated, coiled-coil containing protein kinase (ROCK) [16,17]. TGFβ activates SMAD2/3 and forms complexes with SMAD4, which regulate the transcription of EMT-related target genes, resulting in the repression of epithelial marker gene expression and the activation of mesenchymal gene expression. TGFβ signaling interacts with the WNT and NOTCH pathways.

The EMT is facilitated by oncogenic RAS via the RAS/RAF/MEK/ERK and the RAS/PI3K/AKT/mTOR pathways, which are frequently and abnormally activated in cancer [18]. The canonical WNT-FDZ2 pathway causes disruption of the AXIN/APC/GSK3β complex followed by the nucleic translocation of β-catenin to activate the TCF/LEF family [19]. WNT5a is a prototypical activator of the non-canonical WNT pathway that is associated with the ROR1/AKT/p65 pathway [20]. All of these signaling pathways activate transcription factors, including the SNAIL family SNAI1 (Snail) and SNAI2 (Slug), the zing-finger E-box-binding homeobox factor ZEB1/2, the basic helix-loop-helix factor TWIST, and the TCF/LEF family LEF1, and consequently induce downregulation of adhesion molecules including occludin, ZO1/2, and E-cadherin, and upregulation of mesenchymal molecules including β-catenin, N-cadherin, vimentin, and fibronectin [1,2]. A deubiquitinase, DUB3, which is a target of cyclin-dependent kinase 4/6 (CDK4/6), is essential for the stabilization of SNAI1 [21].

An oncosuppressor TP53 suppresses EMT through activation of the Snail-repressor miR-34 [22] and Slug degradation mediated by MDM2 [23]. Accordingly, TP53 loss and mutation, which are frequently seen in cancer, promote cancer EMT. The EMT signaling upregulates the immune checkpoint molecule PD1 ligand (PDL1, CD274) expression in tumor cells dependent on the PI3K/AKT and MEK/ERK pathways, and PDL1 functionally regulates EMT through the RAS/ERK pathway [24,25].

The EMT-initiated tumor cells produce EMT-inducible molecules to further facilitate EMT in an autocrine manner: for example, chemokine ligand 2 (CCL2) through the Hedgehog signaling pathway [26,27], IL4 through the E2F11/SP3/STAT6 pathway [28,29], and IL35, a member of the IL12 family, through the GP130-STAT1 pathway [30], suggesting a feedback loop for EMT amplification. Particularly, the significant roles of a member of the BM-40/SPARC/osteonectin family follistatin-like 1 (FSTL1) in cancer EMT have been widely demonstrated in many types of cancers, such as melanoma [31], esophageal squamous cell carcinoma [32], head and neck squamous cell carcinoma [33], colorectal cancer [34], and lung cancer [35].

## 3. EMT-Induced CSCs

EMT confers not only high mobility but also stemness including high self-renewability and dormancy, which are representative features of treatment resistance, on tumor cells and special subpopulations that have been strenuously investigated as CSCs [1,36]. The loss and mutation of TP53 intrinsically contribute to the self-renewability through downregulation of the downstream genes, including CDKN1A, PCNA, GADD45, BAX, NOXA, MDM2, and miR-34, followed by upregulation of EMT/CSC-related genes, such as SNAIL and SLUG [37], and pluripotency-related transcription factors, such as OCT3/4, KLF4, SOX2, and c-MYC [38].

Some of the genes are also upregulated by PDL1 induction through BMI1 signaling [39]. Cross-talk among the multiple signaling pathways induces the expression of special molecules, such as CD24, CD44, CD133 (PROM1), CD166 (ALCAM), CD271 (NGFR), epithelial cell adhesion molecule (EpCAM), LGR5 (GPR49), and aldehyde dehydrogenase (ALDH), and the combined markers have been used to define a small subpopulation with CSC-like properties in various types of cancer [36,40]. Although most molecules are only phenotypic markers, some of them functionally regulate the cancer stemness. For example, CD44 plays a key role in the acquisition and maintenance of cancer stemness via binding to hyaluronan [41].

CD24 promotes cell proliferation, invasion, and metastasis through the upregulation of HER2 and EGFR via NF-κB activation [42]. CD133 regulates anti-apoptosis by upregulating FLIP expression through the WNT/β-catenin and PI3K/AKT pathways, and also angiogenesis by inducing vascular endothelial growth factor (VEGF) and IL8 through the WNT/β-catenin pathway [43]. CD166 is a multifunctional molecule that regulates sphere formation and is representative of self-renewability via its homodimerization, anti-apoptosis in cooperation with BCL2, and cell proliferation and invasion through the YAP-TEAD pathway [44,45]. CD271 regulates tumorigenicity, invasion, and metastasis in cooperation with SLUG [46,47].

Quiescent tumor cells in the G0/G1 phase are essentially refractory to the conventional anti-mitotic chemotherapeutics used in clinical therapy [11,48]. Although all tumor cells are likely to have the ability to be dormant and then exit the dormancy to recur [49], the loss and mutation of TP53 profoundly contribute to tumor dormancy by damaging cell cycling [37]. Indoleamine 2,3-dioxygenase (IDO), which is frequently upregulated in various types of cancer, is also known to regulate tumor dormancy. IDO is a cytosolic heme-containing enzyme that degrades tryptophan (which is essential for maintaining physiological and immunological homeostasis) into kynurenine, which triggers G0/G1 cell cycle arrest [50]. Kynurenine also activates a cytosolic transcription factor, aryl hydrocarbon receptor (AhR), followed by the upregulation of IL-6 to maintain IDO expression in an autocrine manner [51]. The TGFβ/WNT-triggered EMT signaling also confers bone metastatic tropism on tumor cells [52,53]. Bone marrow, which is a fertile region containing numerous immune components, such as thrombospondin 1 (TSP1), CXCL12, E-selectin, NOTCH, BMP, and TGFβ, has been considered as a niche for the dormant CSCs [54].

However, accumulating evidence suggests that CSCs also exist in the peripheral blood of cancer patients, and the frequency of the circulating tumor cells (CTCs) is significantly associated with disease stages, metastases, treatment response, and the poor prognosis of cancer patients [55,56].

## 4. Tumor Regeneration after EMT

The dormant CSCs need to reawaken to grow in the metastatic secondary site. MET, the reverse process of EMT, has been believed to be the sequential step in metastatic colonization [57]. MET in the dormant CSCs is triggered by inflammatory stimuli, including neutrophil extracellular traps (NETs) [58,59] and IL35, a novel member of the IL-12 family, released from tumor-associated macrophages (TAMs) within the metastatic site [60]. However, metastatic tumors are likely more aggressive than primary tumors, implying a plasticity mechanism but not a simple reversibility to the original epithelial type.

A strong candidate is polyploidy, where giant tumor cells are generated through mis-regulation of the canonical G1–S–G2–M cell cycle without cell division, followed by rapid proliferation of the progeny cells with genomic instability [6,7,8]. Cell polyploidization is known to be caused by multiple processes, including cell fusion, endoreplication, and ageing-caused senescence, and is now gathering attention as a pivotal role in CSCs since the unrestrained propagation of the progeny cells facilitates tumor evolutional transformation by massively increasing the intra-tumoral heterogeneity and complexity, resulting in refractory cancer.

Polyploid giant tumor cells have been pathologically recognized, particularly in bones, for a long time [61,62], and clinical evidence has shown that the incidence and frequency of such tumor cells are significantly associated with the grade, chemoresistance, and poor prognosis of cancer patients [63,64]. Polyploidization is regulated by AURKA [65], AURKB [66], and c-MYC [67], followed by the degradation and inactivation of TP53, which is important for the cell cycle checkpoint. Interestingly, the transcriptome and proteomic analysis of heart, liver, and placenta cells, albeit non-cancerous cells, revealed that c-MYC interacting genes were associated with EMT- and polyploidy-related genes [68]. This implies that c-MYC may be a key linker between EMT and polyploidy in cancer.

We recently identified IL33 as a key molecule regulating cancer polyploidy. IL33 expression in a nucleus triggers polyploidization following Snail deregulation and TP53 inactivation in bone metastatic tumor cells, and the polyploid giant cells generate small progeny cells in response to treatment stress, showing hyperproliferation in vitro and in vivo [69]. Treatment with chemotherapeutics and ICIs adversely promotes IL33^+^ tumor growth and metastasis through the release of IL33, which systemically expands immunoregulatory ST2^+^ cells in mouse tumor models, as with the unexpected hyperprogression that is sometimes seen in ICI-treated patients [14,15]. The polyploidy mechanisms may be underlying hyperprogression.

A relationship between IL33 and CSCs has also been demonstrated elsewhere. For example, exogenous IL33 stimulation or IL33 overexpression in tumor cells confers stemness on tumor cells through ST2 signaling to activate NANOG, NOTCH, and OCT3/4 in colon cancer [70] and hepatocellular carcinoma [71].

## 5. Immune Supporters for Cancer Metastasis

The intrinsic changes in tumor cells are fostered by numerous components in the host, and the reciprocal evolution raises the possibility of cancer metastasis [9,10].

**Figure 2:**
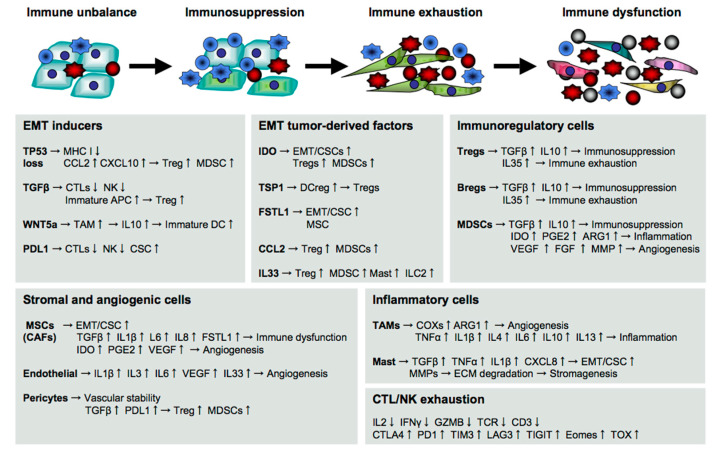
Immune determinants driving cancer metastasis. Cancer metastasis is induced and facilitated by numerous immunological components in the host. The EMT-induced tumor cells produce numerous cytokines and chemokines to induce immune suppression, exhaustion, and dysfunction sequentially toward a successful escape.

Tumor cells first create a permissive environment for the escape by orchestrating immune functions mediated by a variety of cells, such as stromal cells, vascular cells, and immune cells in the host (Figure 2). CSCs are known to metastasize into a bone marrow niche by acquiring bone metastatic tropism through EMT [52,53]. The bone metastasis slowly but seriously damages the immune system through hematopoietic dysfunction in the host.

### 5.1. Immune Evasion at the Beginning of Cancer Metastasis

T cells and natural killer (NK) cells are vital to suppress and eliminate metastatic and malignant tumor cells. Tumor-specific cytotoxic T cells (CTLs) are generated and activated through interaction between the T-cell receptor (TCR) and the major histocompatibility complex (MHC)–peptide complexes expressed on antigen-presenting cells (APCs), including dendritic cells (DCs) and macrophages [72]. Stable engagement with costimulatory molecules, including CD80, CD83, and CD86, is necessary for intensification of the TCR/MHC/peptide stimulatory signals to induce potent CTLs that recognize the target tumor cells through MHC I [72].

However, the downregulation or loss of MHC I expression is frequently seen in CSC-like tumor cells due to TP53 decrease [73], histone deacetylase (HDAC) mutation [74], or EMT signaling [75]. This intrinsically enables tumor cells to evade CTL attacks. The loss and mutation of TP53 in tumor cells also induce the production of various chemokines, such as CCL2 and CXCL10, and immunosuppressive regulatory T cells (Tregs) and myeloid-derived suppressor cells (MDSCs) are recruited into the tumor microenvironment [76,77].

EMT inducers also create a tolerant environment to facilitate the tumor escape. For example, TGFβ suppresses cytotoxic functions of CTLs and NK cells directly by reducing the expression of perforin, granzyme B, and NKG2D, and indirectly by expanding Tregs and immature APCs [52,78]. WNT5a stimulates TAMs to release IL10 through the TLR/MyD88/p50 pathway [20]. The released IL10 suppresses the maturation of DCs by reducing the expression of T-cell stimulatory molecules [79], and also intensifies cancer stemness through the JAK1/STAT1/NFkB pathway [80].

### 5.2. Immunosuppression during Cancer Metastasis

EMT signaling also induces the production of numerous cytokines, which not only suppress immune responses against cancer but also further facilitate EMT as a feedback loop. For example, PDL1 expression in CSCs protects from immune attack by braking the activation signaling pathways in anti-tumor effector cells, such as T cells and NK cells, via programmed cell death 1 (PD1) signaling [81,82].

CSC-producing IDO degrades tryptophan into kynurenine, which activates the cytosolic transcription factor AhR that is widely expressed in immune cells [50]. The AhR activation not only suppresses cytotoxicity, proliferation, and survival of CTLs and NK cells, but also generates Tregs and MDSCs, resulting in impairment of anti-tumor immunity [58,83]. Snail-induced thrombospondin-1 (TSP1) promotes tumor EMT in an autocrine manner [84,85], and indirectly through the generation of Treg-inducible regulatory DCs (DCreg) [86].

Snail-induced FSTL1 directly damages CTL induction [35], and indirectly through the induction of pluripotent mesenchymal stem/stromal cells (MSCs), which not only generate dysfunctional CD8^low^ CTLs via ALCAM signaling but also further promote tumor metastasis, especially to the bone marrow [31]. FSTL1 is widely known as an inflammatory and pathogenic molecule associated with many diseases, such as rheumatoid arthritis [87,88], obesity [89], and cancer [90]. In cancer, FSTL1 plays a critical role in tumor malignancy, including tumor proliferation, clonogenicity, invasion, metastasis, self-renewal, and chemoresistance, in various types of cancer, such as breast cancer [31], lung cancer [35], colorectal cancer [34], head and neck cancer [33], and esophageal squamous cell carcinoma [32]. CSCs also produce various chemokines, such as CCL2 (MCP1), to recruit immunosuppressive CCR2^+^ cells, including Tregs and MDSCs [31,91], and CXCL1/2 to recruit CXCR2^+^ MDSCs that enhance tumor survival [92,93].

Tregs play a central role in the maintenance of self-tolerance and homeostasis and, thus, suppress the immune responses against self-born cancer cells [94]. In tolerance mechanisms, cytotoxic T-lymphocyte-associated protein 4 (CTLA4) is immediately upregulated to brake the self-reactive responses by binding to ligands, including CD80 and CD86 [95,96]. After activation, PD1 is upregulated to attenuate TCR signaling by binding to the ligand PDL1 [97,98]. Tregs are a heterogenous population of immunosuppressive T cells expressing tissue- or function-specific transcription factors, such as GATA3 and STAT3, along with FOXP3, which is a hallmark transcription factor of Tregs [78]. Tregs produce various cytokines, such as TGFβ and IL10, which induce immunosuppression and tumor aggravation as described above [99]. IL35 is a current topic as a Treg-produced effector molecule in cancer metastasis. IL35 promotes tumorigenesis and tumor transendothelial extravasation by enhancing ICAM1 expression through the gp130- IL-12Rβ2 pathway [30,100] and also robustly induces multiple immune checkpoint molecules, such as PD1, T-cell immunoglobulin mucin 3 (TIM3, HAVCR2), lymphocyte-activation gene 3 (LAG3), and T-cell immunoreceptor with Ig and ITIM domains (TIGIT), in T cells and NK cells, resulting in immune exhaustion and dysfunction [100,101].

MDSCs are a heterogeneous population composed of mononuclear and polymorphonuclear myeloid cells, which produce or express various immunomodulatory molecules, such as TGFβ, IL10, IDO, PGE2, ARG1, and PDL1, to promote cancer metastasis directly and indirectly by suppressing anti-tumor immune responses as described above [102,103]. Accumulating evidence suggests a potential role of regulatory B cells (Bregs) in cancer metastasis. Bregs increase not only in primary tumor tissues [104] but also in the metastatic bone tissues of patients [105] and produce immunosuppressive molecules, such as TGFβ, IL10, and IL35, which promote tumor progression and metastasis directly and indirectly as described above [104,106].

MSCs are multipotent stromal cells with high migratory and immunosuppressive properties, and they are able to differentiate into a variety of mesenchymal lineages, such as adipocytes, osteocytes, chondrocytes, fibroblasts, and pericytes [107]. MSCs are originally silent but become aggressive after receiving activation signals from tumor-derived pro-inflammatory cytokines, such as TNFα and IL1β, and/or ligation of the TLRs, such as TLR2, TLR3, and TLR4, that are expressed on MSCs [108,109]. The activated MSCs robustly support tumor progression and metastasis directly by inducing EMT, and indirectly through the production of numerous immunomodulatory molecules, such as TGFβ, PGE2, IL6, IL8, VEGF, TNFα, IL1β, IDO, and FSTL1, to generate and expand Tregs, MDSCs, and DCregs [109,110].

After reawakening from dormancy, CSCs increasingly orchestrate immunosuppression. At the generation of the progeny cells in response to treatment stress, polyploid giant cells abundantly produce IL33, which expands ST2^+^ cells composed of Tregs, MDSCs, mast cells, and type 2 innate lymphoid cells (ILC2s) to further facilitate their own escape [69]. Although the role of ILC2s is still controversial in cancer, many studies have demonstrated a relationship with cancer metastasis and immunosuppression. For example, as well as MSCs, ILC2s become suppressive upon activation, and the activated ILC2s produce various cytokines, such as IL4, IL5, IL9, and IL13, to impair the anti-tumor immunity, particularly of NK cells [111,112]. Stimulation with TGFβ, IL1β, and IL23 induces the conversion of ILC2s into RORγt^+^ Th17 cells, which are deeply associated with inflammatory and autoimmune diseases, including cancer [113,114].

### 5.3. Stromagenesis and Angiogenesis for Cancer Metastasis

Tumor-surrounding stroma is a fertile environment rich in numerous components and composed of extracellular matrix and multiple types of immunoregulatory cells, such as MSCs, fibroblasts, pericytes, endothelial cells, and immune cells, which provides oxygenation and nutrition to maintain and promote tumor development, progression, invasion, and metastasis through stromagenesis and angiogenesis [115].

MSCs promptly migrate into tumor sites in response to chemokines, such as CCL2, CCL5, CXCL12, and CXCL16, and are expanded by cytokines, such as TGFβ, VEGF, FGF, and IGF, all of which are released from the tumor milieu [116]. The tumor-activated MSCs orchestrate stromagenesis and angiogenesis for promoting tumor invasion and metastasis directly and indirectly through both the differentiation into vascular cells and the induction of immunosuppression as described above [109,110]. Cancer-associated fibroblasts (CAFs) are immunosuppressive and tumor-promotive in the same way as MSCs [117,118].

Mast cells are also a key contributor to stromagenesis and angiogenesis. Mast cells are recruited by CCL2, CXCL1, CXCL10, and CXCL8/IL8 and are activated by SCF, VEGF, angiopoietin, PGE2, LTB4, and osteopontin released from the tumor milieu [119], and the activated mast cells produce TGFβ, TNFα, IL1β, and CXCL8 to induce EMT as well as MMP9 to induce degradation of the extracellular matrix for tumor invasion and metastasis [120].

Endothelial cells lining the inner walls of blood vessels play a key role in angiogenesis through the development of new blood vessels from pre-existing vessels, and protect CSCs by producing a variety of immunomodulatory and angiogenic cytokines, such as IL1, IL3, IL6, IL33, and VEGF [121,122]. The VEGF family is well known as a potent stimulator of blood vessel formation [123].

IL33 also greatly contributes to angiogenesis through the recruitment and activation of not only immunosuppressive ST2^+^ MDSCs that produce VEGF, FGF, and MMP9 [102] but also inflammatory ST2^+^ cells, including tumor-associated macrophages (TAM) and mast cells [119,124]. Pericytes typically maintain vascular stability and homeostasis by wrapping around endothelial cells and by depositing extracellular matrix [125,126]. After activation, however, pericytes acquire immunosuppressive properties through increases in their PDL1/2 expression and TGFβ production [127].

### 5.4. Immune Exhaustion and Dysfunction for Cancer Metastasis

Inflammatory cells also seriously damage the immune system. In particular, myeloid cells including TAMs and activated mast cells play a crucial role in chronic inflammation through the production of a variety of inflammatory molecules, such as cyclooxygenases (COXs, PTGSs), prostanoids, ARG1, TNFα, IL1β, IL4, IL6, IL10 and IL13 [120,128]. Persistent and strong stimulation with these factors further upregulates IDO expression, and widely impairs innate and acquired immunity [58].

Immune exhaustion and dysfunction of cytotoxic T cells and NK cells are fatal to the host with cancer, since multiple immune inhibitory molecular expressions, including CTLA4, PD1, TIM3, LAG3, and TIGIT, are abundantly induced for braking of the activation signals followed by a decrease in anti-tumor effector molecules, such as IL2, IFNγ, TNFα, and granzyme B (GZMB) [129]. CTLA4 is an innate brake to suppress the initial T-cell activation, and then PD1 expression is induced and sustained upon activation followed by the downregulation or degradation of TCR, which is essential for the generation and activation of potent CTLs [130].

LAG3 expression widely suppresses anti-tumor immunity directly by impairing T/NK cells, and indirectly through impeding CD4^+^ T-cell functions via binding to MHC class II (MHC II) with a higher affinity than CD4 [131]. TIGIT expression impairs T/NK cells via binding to CD155 (PVR) and CD112 (Nectin2) expressed in myeloid cells and tumor cells [132]. Exhaustion and dysfunction of NK cells present serious damage in cancer immunotherapy, as CTLs sometimes miss tumor cells due to the loss of MHC on tumor cells as described above [133].

Recently, an HMG-box transcription factor, thymus high mobility group box protein (TOX), was identified as a key regulator of T-cell exhaustion accompanied by constitutive PD1 expression [134]. A recent study demonstrated that TOX orchestrates immune inhibitory signals, not only PD1 but also other immune checkpoint molecules, such as CTLA4, TIM3, and TIGIT, in CD8^+^ T cells [135], likely because TOX binding to PD1 promotes the endocytic recycling of PD1 to maintain abundant PD1 expression on the cell surface [136]. CD101 (IGSF2) was identified as a possible marker to distinguish transitionally exhausted T cells, which still exert anti-tumor activities by invigoration from terminally exhausted T cells [137].

## 6. Treatments for Cancer Metastasis

**Figure 3:**
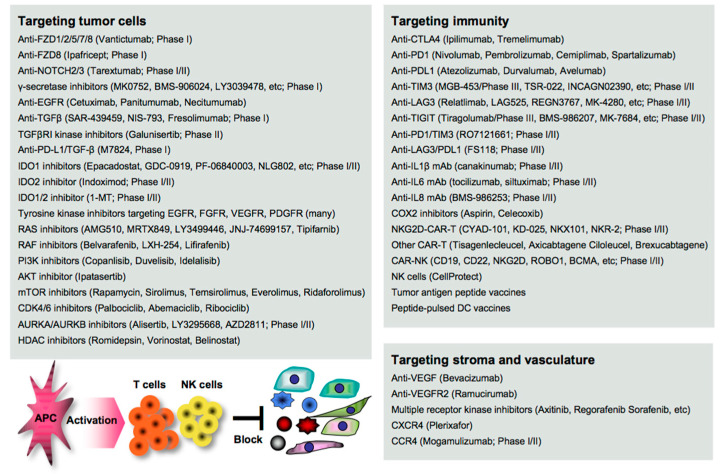
Treatments targeting the oncoimmunological determinants driving cancer metastasis. Numerous agents targeting the cancer metastatic cascade have been pharmaceutically developed, although many clinical evaluations are still underway. The proper combination of certain agents may successfully treat cancer patients.

Disruption of the cancer metastatic cascade is a promising strategy for treating cancer. Numerous agents targeting oncoimmune drivers, including small molecule inhibitors, antibodies, and genetically modified cells, have been pharmaceutically developed in clinical settings, as also reviewed elsewhere [138]. However, most of the clinical evaluations are still underway, and the therapeutic efficacy reported so far is limited to a subset of patients. Here, we summarize the recent advances in the development of agents as guidance for designing more effective treatment regimens in clinical settings (Figure 3).

### 6.1. Targeting EMT/CSC Inducers

EMT stimuli simultaneously create a permissive environment for tumor escape in the host; thus, blocking the initiation of EMT appears to be a great rationale for the clearance of the complicated consequences. Vantictumab is an inhibitory mAb specific for the WNT receptors FZD1/2/5/7/8, which has been evaluated in a phase I study for solid tumors [139]. Ipafricept is a recombinant fusion protein with the extracellular domain of FZD8 and has been evaluated in combination with chemotherapy in a phase I study for advanced solid tumors [140]. Tarextumab (OMP-59R5) is an anti-NOTCH2/3 mAb and has been evaluated in phase I/II studies for solid tumors, including pancreatic cancer [141,142] γ-secretase is essential for NOTCH activation, and thus many inhibitors, including MK0752 [143], BMS-906024 [144], LY3039478 [145], RO4929097 [146], and LY900009 [147], have been evaluated in combination with/without chemotherapy in phase I studies for advanced or metastatic cancer.

Anti-EGFR mAbs (cetuximab, panitumumab, and necitumumab) have been clinically approved in combination with/without chemotherapy for various types of cancer [148,149]. Tyrosine kinases are essential for the activation of the signal transduction of receptors, and numerous inhibitors have been clinically developed and approved targeting EGFR (gefinitib, erlotinib, afatinib, simotinib, etc.), VEGFR (sunitinib, lenvatinib, vandetanib, etc.), FGFR (erdafitinib, infigratinib, pemigatinib, rogaratinib, etc.), PDGF (imatinib), and multiple receptors (axitinib, cabozatinib, ponatinib, regrorafenib, amcasertib, etc.) [150,151,152].

Blocking TGFβ and IDO is expected to widely organize the tumor environment, as both molecules have a broad range of biological activities on both tumor cells and host immunity as described above. Several anti-TGFβ mAbs (SAR-439459, NIS-793, and fresolimumab) have been evaluated in phase I studies for advanced solid tumors [153]. Galunisertib (LY2157299) is a small molecule inhibitor of TGFβ receptor I (TGFβRI) kinase for SMAD2 phosphorylation, and clinical outcomes have been reported in phase II studies for recurrent glioblastoma [154] and advanced hepatocellular carcinoma [155].

These TGFβ-targeting mAbs and inhibitors are often combined with anti-PD1/PDL1 therapy in clinical trials, as synergistic efficacy has been reported in mouse models [156]. M7824 is a bifunctional anti-PDL1/TGF-β trap fusion protein, which not only efficiently reverts the mesenchymalization of tumor cells, but also activates CTLs and NK cells [157], and many clinical trials have been conducted. Inhibitors targeting IDO1 (epacadostat, GDC-0919, PF-06840003, NLG802, SHR9146, and linrodostat), IDO2 (indoximod), or both (1-MT) have been evaluated in combination with chemotherapy and/or ICI therapy in phase I/II studies for solid tumors and peritoneal cancer [158]. In the phase 3 ECHO-301/KEYNOTE-252 study, however, the addition of epacadostat to pembrolizumab showed no significantly greater clinical benefit on overall survival and progression-free survival compared with pembrolizumab monotherapy in patients with unresectable or metastatic melanoma [159]. A reason may be that IDO and PDL1 expressions are generally low in melanoma tissues, as shown in open databases such as the Human Protein Atlas (http://www.proteinatlas.org/). The combination regimen may be effective in gastrointestinal cancer with higher expressions of both molecules.

RAS/RAF promotes the EMT through the MEK/ERK and the PI3K/AKT/mTOR signaling pathways [18], and thus numerous inhibitors have been clinically developed and approved targeting RAS (AMG510, MRTX849, LY3499446, JNJ-74699157, and tipifarnib), RAF (belvarafenib, LXH-254, and lifirafenib), PI3K (copanlisib, duvelisib, and idelalisib), AKT (ipatasertib), and mTOR (rapamycin, sirolimus, temsirolimus, everolimus, and ridaforolimus) for treating cancer patients [18,160].

Targeting the cell cycle-related CDK4/6/DUB3 pathway is also expected to contribute to the suppression of cancer metastasis, as blocking CDK4/6 and the related DUB3 is effective in mouse metastasis models [161,162]. CDK4/6 inhibitors (palbociclib, abemaciclib, and ribociclib) have been clinically approved and used in combination with/without hormone therapy for treating metastatic breast cancer [163]. Targeting polyploidy-related aurora kinases, including AURKA and AURKB, may also be useful for suppressing cancer metastasis as reported in mouse studies [164].

Many AURKA/AURKB inhibitors (alisertib, LY3295668, AZD2811, etc.) have been pharmaceutically developed [165]; however, most of the clinical trials failed. Six phase I/II studies have been now conducted in combination with chemotherapy and/or ICI therapy for various types of cancer (http://clinicaltrials.gov). The signaling network interacting with other signaling pathways is extremely complicated, and a number of feedback loops create refractory cancer. Therefore, combinations with certain agents could be better for treating cancer patients.

### 6.2. Targeting Stromagenesis and Angiogenesis

Blocking stroma-regulating drivers is also expected to impede tumor growth and dissemination. Despite the significant roles of MSCs and CAFs in cancer metastasis, directly targeting them is impractical because the precise characteristics are still obscure. Instead, blocking MSC/CAF-produced chemokines and the receptors may be an alternative approach. Many inhibitors have been pharmaceutically developed, and some targeting CXCR4 (plerixafor), CCR4 (mogamulizumab), and CCR5 (maraviroc) have been clinically approved, although only plerixafor is applicable for treating cancer, non-Hodgkin’s lymphoma, and multiple myeloma [166,167]. Mogamulizumab has now been evaluated in combination with anti-PD1 mAb (ClinicalTrials.gov Identifier: NCT03309878) or IL15 (ClinicalTrials.gov Identifier: NCT04185220) in phase I/II study for relapsed or refractory lymphoma and leukemia.

In contrast, targeting angiogenesis and vascularization has been progressed over time, and many small molecule inhibitors have been clinically approved targeting VEGF (bevacizumab), VEGFR2 (ramucirumab), and multiple receptors, including VEGFR, PDGFR, FGFR, KIT, RET, and FLT3 (axitinib, cabozantinib, nintedanib, pazopanib, regorafenib, sorafenib, and sunitinib) [148,149]. However, the anti-tumor efficacy of monotherapy is modest and limited, and combination regimens with other agents, including ICIs and chemotherapeutics, have now been evaluated in many clinical trials.

### 6.3. Targeting Immune Determinants

Immune mediators participate in the mechanisms underlying cancer metastasis. Blocking immune inhibitory pathways is important for inducing potent anti-tumor immunity, and targeting CTLA4/PD1 signaling has attracted great attention in cancer therapy [130]. There are many clinically approved mAbs targeting CTLA4 (ipilimumab and tremelimumab), PD1 (nivolumab, pembrolizumab, cemiplimab, and spartalizumab), and the ligand PDL1 (atezolizumab, durvalumab, and avelumab), and targeting the PD1-PDL1 axis has been widely recognized as a successful strategy for treating advanced and metastatic cancer, despite the clinical outcomes being extremely limited [12].

Biomarkers that predict potential responders to anti-PD1/PDL1 therapy have been strenuously investigated by analyzing patient-derived specimens using advanced technology, and several biomarkers, including PDL1 expression, microsatellite instability (MSI), and mutation burden (the number of non-synonymous single nucleotide variants) in tumor cells have been identified [168,169]. However, these are not necessarily correlated with clinical outcomes, and more precise and accurate biomarkers are still being explored in clinical settings. Combination regimens that potentially optimize the ICI efficacy have also been strenuously investigated, and numerous clinical trials with a variety of agents, such as small molecule inhibitors, ICIs, and vaccines, have been conducted around the world [170].

Targeting inflammatory mediators is also important for alleviating tumor aggravation and immune-related adverse events, including autoimmunity, which is frequently found in ICI therapy [13]. In general, inflammatory mediators have been pharmaceutically targeted primarily for treating other inflammatory diseases, such as rheumatoid arthritis and pulmonary disease, so far. However, several inhibitory mAbs have been clinically evaluated for treating cancer by targeting IL1β (canakinumab) [171], IL6 (tocilizumab, siltuximab) [172], and IL8 (BMS-986253) [173] in combination with/without other agents, such as chemotherapy, anti-HER2 mAb, or anti-PD1 mAb, in phase I/II trials.

COXs are representative inflammatory mediators that produce eicosanoids, such as PGE2 (mainly produced by COX2) and TXA2 (mainly produced by COX1) from arachidonic acid, and are highly and frequently expressed in both myeloid cells and tumor cells [174]. A number of preclinical studies demonstrated the therapeutic efficacy induced by a COX1/2 inhibitor aspirin on cancer metastasis by inhibiting platelet aggregation, endothelial activation, tumor cell adhesion to the endothelium, the recruitment of myeloid cells, and the EMT of tumor cells [175]. The clinical significance of aspirin use has also been demonstrated in colorectal cancer patients, particularly with PDL1^low^ tumors [176,177]. However, most of the clinical studies are retrospective, and the therapeutic efficacies of COX inhibitors remain to be determined in clinical settings [178]. Interestingly, one study reported that treatment with a CDK4/6 inhibitor palbociclib suppressed COX2/PGE2 by the repression of c-JUN expression, resulting in the suppression of cancer metastasis in mouse breast cancer models [161]. Persistent and strong stimulation with inflammatory mediators induces exhaustion and dysfunction in anti-tumor effector cells, accompanied by the upregulation of multiple immune checkpoint molecules, including TIM3, LAG3, and TIGIT; therefore, blocking these negative signals is expected to reinvigorate the immune fighters against cancer [129]. Many inhibitory mAbs targeting TIM3 (TSR-022, MGB-453, INCAGN02390, Sym023, and BGB-A425), LAG3 (Relatlimab, LAG525, REGN3767, MK-4280, FS118, Syn-022, and TRS-003), and TIGIT (tiragolumab, BMS-986207, MK-7684, AB154, ASP8374, and COM902) have been clinically evaluated in combination with/without other agents, such as chemotherapeutics and ICIs in phase I/II trials and MGB-453 and tiragolumab in phase III trials (ClinicalTrials.gov: https://clinicaltrials.gov/).

MGB-453 has been evaluated for chronic myelomonocytic leukemia (ClinicalTrials.gov Identifier: NCT04266301), and tiragolumab has been evaluated in combination with/without chemotherapeutics or atezolizumab for esophageal squamous cell carcinoma (ClinicalTrials.gov Identifier: NCT04543617) and small cell lung cancer (ClinicalTrials.gov Identifiers: NCT04256421, NCT04294810). In a randomized phase II CITYSCAPE study (*n* = 135), combinations with Tiragolumab and atezolizumab showed a clinical benefit on the overall response rate (37% versus placebo 21%) and progression-free survival (5.5 months versus placebo 3.88 months) in non-small cell lung cancer [179]. FS118 is a LAG3/PDL1- bispecific mAb that was evaluated in a phase I study for advanced and/or metastatic cancer (ClinicalTrials.gov Identifier: NCT03440437), and RO7121661 is a PD1/TIM3-bispecific mAb that was evaluated in a phase I study for advanced and/or metastatic solid tumors (ClinicalTrials.gov Identifier: NCT03708328).

The removal of bad and negative factors on immunity is a promising approach to cancer treatment. However, the induction and activation of anti-tumor immune responses is a principle of immunotherapy of cancer, and active immunotherapy could pave the way to success in strengthening the anti-tumor immune power. Despite the rare success of active immunotherapy with classical immunomodulatory agents, including whole tumor vaccines, DC vaccines, tumor antigen peptides, and viral vectors, tumor antigens have been re-focused as a useful tool to stimulate immunity, since high mutations in tumor cells, including CSCs, are believed to generate more immunogenic tumor antigens—so-called neoantigens [180].

Next generation sequencing, including exome and RNA sequencing, combined with advanced bioinformatics technology enabled researchers to identify and predict neoantigens and numerous peptide vaccines targeting neoantigens (KRAS, DNAJB1-PRKACA, IDH1R132H, AE37, K27M, etc.), and peptide-pulsed DC vaccines have been clinically evaluated in combination with other treatments, such as chemotherapy and ICI therapy, for various types of cancer [181].

However, active immunotherapeutic strategies often fail, as CTLs are unable to recognize CSC-like tumor cells with the MHC loss caused by a TP53 decrease [73], HDAC mutation [74], or EMT signaling [75]. HDAC inhibitors have been pharmaceutically developed not only to enhance MHC I expression and immunogenicity, but also to suppress cancer EMT [182,183], and four HDAC inhibitors have been clinically approved for treating lymphoma (romidepsin, vorinostat, and belinostat) and myeloma (panobinostat). Combinations with anti-PD1/PDL1 therapy may be congenial to the therapy, as treatment with HDAC inhibitors also upregulates PDL1 expression in tumor cells [184].

However, anti-tumor effector cells are frequently impaired in cancer patients, and this leads to innate resistance to immunotherapy. To overcome this problem, T cells and NK cells have been genetically engineered to strengthen the potency, including the proliferation, survival, and infiltration into tumor tissues for solid tumors [185]. Particularly, T cells that are genetically engineered to express chimeric immunoreceptors (CD3ζ, CD28 and/or 4-1BB, etc.) and so-called CAR-T cells have attracted attention as a promising cell medicine in cancer therapy, and three CAR-T products (tisagenlecleucel, axicabtagene ciloleucel, and brexucabtagene autoleucel) have been clinically approved for lymphoma treatment [186].

Despite success in the treatment of hematological malignancies, therapeutic efficacy is extremely limited in the treatment of solid tumors. As a breakthrough to the treatment of solid tumors, NKG2D expressed in NK cells and CTLs has been recently studied, since NKG2D signaling activates anti-tumor effector cells via binding to the ligands (MICA/MICB, ULBP, RAE1, etc.) that are frequently overexpressed in tumor cells [187]. NKG2D-CAR-T cells (CYAD-101, KD-025, NKX101, and NKR-2) have been clinically evaluated in combination with chemotherapy in phase I/II studies for relapsed or refractory solid tumors (ClinicalTrials.gov Identifiers: NCT03692429 and NCT04550663).

In the phase I study, however, no objective responses were observed due to the limitation of the expansion and persistence of the transferred CAR-T cells in the patients, albeit with no dose-limiting toxicities [188]. Further improvement of the CAR-T design may be needed for success. On the other hand, a growing number of studies have demonstrated the importance of NK cells in EMT/CSC-targeting therapy, as NK cells show cytotoxity in the absence of MHC and antigen presentation [189,190]. Many clinical trials with NK cells have been conducted in combination with/without other agents, and CellProtect has been recently approved as an orphan drug for treating multiple myeloma. CAR-NK cells have also been pharmaceutically developed for various types of cancer, including pancreatic cancer [191].

## 7. Conclusions

Cancer metastasis has been intensely investigated through genomic and oncoimmunological approaches in recent years, and the vague landscape of the oncoimmune metastatic cascade has been increasingly clarified. To overcome the limited efficacy of conventional treatments, multi-acting anti-cancer agents targeting multiple signaling pathways, or ICIs targeting different immune checkpoint pathways, have been continuously generated, and dozens of clinical trials have been developed all over the world.

However, strong anti-tumor effects simultaneously generate high-grade toxicity, including autoimmunity, and a significant proportion of patients acquire resistance to the treatment. Novel therapeutic strategies are still needed to disrupt the vicious spiral of tumor–immunity aggravation. To further deepen the understanding of both the entire metastatic cascade and the clinical implementations, it may be helpful to design an ideal treatment regimen, particularly in the context of combinations with different agents.
